# Adaptive plant traits under anthropogenic burning regimes: A database for UK heath and mire plant species

**DOI:** 10.1002/ajb2.70090

**Published:** 2025-09-03

**Authors:** Kimberley J. Simpson, Claire M. Belcher, Sarah J. Baker

**Affiliations:** ^1^ School of Biosciences University of Sheffield Sheffield South Yorkshire UK; ^2^ Botany Department Rhodes University Makhanda South Africa; ^3^ wildFIRE Lab University of Exeter Exeter UK

**Keywords:** adaptive traits, heathlands, land management, moorlands, peatlands, plant functional traits, prescribed fire, rotational burning, temperate ecosystems

## Abstract

**Premise:**

Humans have used fire to manage landscapes for millennia, but this use of fire is declining in many ecosystems. Understanding how plants respond to these changes is key to predicting ecosystem resilience and impacts on services such as biodiversity and carbon sequestration. However, many ecosystems lack data on plant fire responses. A solution is to infer these responses through studying functional traits that offer fitness benefits under fire by enabling species to resist fire, or persist through fire, by resprouting or recruitment from stored seed banks or dispersed seed. Studying these traits helps predict species performance under fire.

**Methods:**

We used a trait‐based approach to create a database of fire‐relevant traits and fire responses for vascular and nonvascular plant species in UK heath and mire ecosystems managed by fire. By reviewing the available literature, we collected data on traits adaptive under frequent burns for 153 plant taxa that could then be used to make predictions about fire effects.

**Results:**

Of the 153 taxa assessed, 97% had fire‐adaptive traits; 149 taxa had resistance and/or persistence traits, enabling survival or regeneration after fire. Additionally, 20 taxa showed fire‐enhanced recruitment, while only four lacked traits aiding survival during or after fire.

**Conclusions:**

The database created here is designed to be a resource for those who study and manage heath and mire ecosystems. The use of fire in areas where heath is underlain by peat is much debated, and this database can inform how changing or removing the use of fire will impact plant communities.

For millennia, humans have used fire as a tool to manage landscapes, applying it at specific times and intervals to achieve various goals, including enhancing productivity (Goren‐Inbar et al., 2004; Bowman et al., 2011; Roebroeks and Villa, 2011). However, the use of fire to manage some ecosystems has decreased over recent decades, largely due to the perception that fire is always an ecologically damaging process (Davies et al., 2016). For example, the Brazilian Cerrado savanna has been subject to periods of “zero‐fire” policy over the past few decades (Franke et al., 2024). Similarly, the use of fire in ecosystems in the UK, particularly heaths and mires, which have historically been managed by fire, is becoming a topic of considerable debate (Davies et al., 2016). This push toward the reduction or removal of fire management practices is set against a context of increasing wildfire occurrence and severity, where climate change is causing more frequent and intense episodes of fire weather (i.e., conditions favorable to the ignition and spread of fire; Jones et al., 2022), causing the global transformation of wildfire activity (Hantson et al., 2024). Changes in anthropogenic burning practices likely interact with the rising wildfire risk because the reduced use of prescribed burns allows vegetation fuels to accumulate, increasing the risk of more intense and damaging wildfires (Russell‐Smith et al., 2013; Davis et al., 2024; Kreider et al., 2024). Together, changes to anthropogenic burning practices and wildfire regimes (i.e., the typical frequency, intensity and seasonality of fire in a place) result in plants facing a different fire regime than previously experienced, which may have negative implications for plant survival, reproduction, and persistence (Archibald et al., 2018; Grau‐Andrés et al., 2024, and references within).

Understanding how plants respond to changing fire regimes, be they natural or human driven, is therefore key to predicting the resilience of ecosystems to such changes, as well as impacts on ecosystem services such as biodiversity and carbon sequestration. In fire‐prone ecosystems, the impacts of fire and changing fire activity on the species present is well studied (e.g., the Mediterranean Basin, Tavşanoğlu and Pausas, 2018; the Cerrado, Fidelis and Zirondi, 2021; Zupo et al., 2021; Rodrigues and Fidelis, 2022). However, in other ecosystems, particularly those that are not historically fire‐prone but where climate‐change‐induced increases in fire occurrence are occurring (e.g., temperate and boreal ecosystems, tropical rainforests), plant species responses to fire are understudied, therefore limiting our understanding of the resilience of these ecosystems to fire‐regime changes.

To overcome a paucity of data relating to plant species responses to fire, one approach is to infer these responses through the study of functional traits. Functional traits, which encompass morphological, physiological, and phenological characteristics, determine the response of plant species to environmental factors and disturbances (Westoby and Wright, 2006; Violle et al., 2007), and as such, trait‐based approaches are widely used in ecological and evolutionary research, including questions relating to fire (Noble and Slatyer, 1980; Pausas et al., 2004; Keith et al., 2007; Simpson et al., 2021; Plumanns‐Pouton et al., 2024). A number of functional traits that provide fitness benefits in a given fire regime (i.e., adaptive traits; Dobzhansky, 1956) have been identified in plant taxa from fire‐prone ecosystems around the world (e.g., Clarke et al., 2013; Pausas et al., 2017; Lamont et al., 2020; Pausas and Lamont, 2022; Pausas and Keeley, 2023). Traits that enhance fitness through fire may do so by providing resistance to the effects of fire (resistance traits; Rowe 1983) or the ability to regenerate post‐fire after the death or top‐kill of standing individuals (persistence traits). Persistence can be achieved by several mechanisms: resprouting from surviving buds (Clarke et al., 2013) or the recruitment of a new generation from seed that survives fire (i.e., a fire‐resistant seed bank) or by seed that disperses into fire‐affected areas (the exogenous recolonization strategy sensu Pausas, 2019). Data on traits likely to be adaptive under a fire regime are available through a variety of sources (e.g., journal articles, theses, books, governmental reports, trait databases, species descriptions) and may be used collectively to infer the plant responses to fire when that information is lacking.

Here, we adopted a trait‐based approach to create a database of fire‐relevant traits and fire responses in plant communities of heath and mire ecosystems, which incorporate heathlands and peatlands, in the United Kingdom. These open ecosystems are dominated by dwarf shrubs and herbaceous plant species and are largely the result of forest clearance and domestic livestock grazing thousands of years ago (Simmons, 2019). Today, they have a high amenity value and provide a number of environmental and economic services, such as carbon storage, drinking water provision, and grazing land (Chapman et al., 2009; Martin‐Ortega et al., 2014). The high flammability of vegetation in these landscapes, with a high fine‐fuel load that dries readily under warm and dry conditions, means that fire has been an important and effective tool in their management over centuries. Indeed, these flammable plant communities fuel the largest proportion (>60%) of area burned in wildfires in the United Kingdom, with over 2000 ha of heathland and bogland burning annually on average (Belcher et al., 2021). However, the use of fire on these landscapes as a management tool has been a topic of considerable debate (Davies et al., 2008; Harper et al., 2018), especially in those habitats underlain by peat due to the potential ignition risk to peatland carbon stocks, pollution, and negative effects on biodiversity. These concerns have led to a ban on burning vegetation that sits on top of deep peat (defined as more than 40 cm deep; DEFRA, 2021) and has led to several large landowners ending this practice (Doward, 2020). However, little consideration has been given to the impacts of the cessation of prescribed burns on plant communities that have experienced and have been shaped by this regular application of fire. In addition, a decline of fire use, when alternative vegetation management practices are not implemented, threaten the conservation of these ecosystems and increases the risk of severe wildfires (Allen et al., 2016).

To develop an effective and sustainable management strategy for these ecosystems, we need an understanding of how the plant species present in these communities are affected by fire. Building this understanding requires a trait‐based approach, because although botanists and ecologists have long investigated the effects of prescribed burning on floristic changes and community succession in upland habitats (e.g., Mallik and Gimingham, 1983; Hobbs and Gimingham, 1984), the information that has been produced has tended to be focused on a few sites and has not captured all species that might be affected. The database presented here on fire‐relevant traits for 153 native UK plant taxa will be useful not only for developing effective management strategies for these ecosystems to future proof and enhance their biodiversity, but also to predict how they may respond to enhanced fire risk due to climate change.

## MATERIALS AND METHODS

### Ecosystem selection

**Figure 1:**
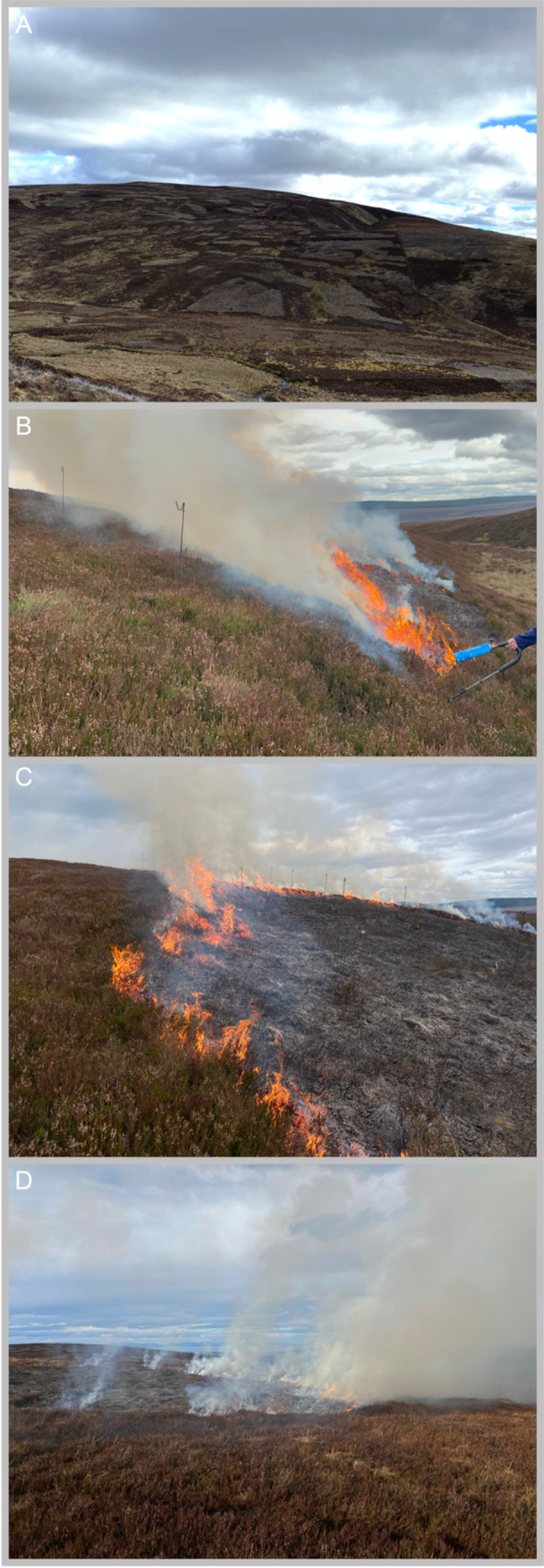
Typical moorland dominated by heather (*Calluna vulgaris*) in the UK showing a mosaic of different aged heather achieved by rotational, prescribed burning (A). (B) Ignition and (C, D) typical behavior of a prescribed fire. Photo credit: C. Belcher.

To create a database of fire‐relevant traits for native UK plant species subject to anthropogenic burning regimes, we focused on species that inhabit ecosystems where fire is purposefully and regularly applied to the landscape. Such ecosystems are classified as heaths and mires in the United Kingdom by the Joint Nature Conservation Committee's National Vegetation Classification (Rodwell, 1991). These vegetation communities are burned using practices that have a range of names including swailing, controlled burning, rotational burning and muirburn, which are largely similar in their approach, but also prescribed fires that are designed for a more specific purpose. (Belcher and colleagues defined these terms in detail [2021, p. 40].) These ecosystems cover a spectrum of environmental conditions reflecting the north–south temperature and east–west moisture gradients across the United Kingdom, and a range of altitudes. In upland settings, habitats dominated by heather (*Calluna vulgaris*) have been traditionally managed by burning different patches of landscape on a rotation to create a mosaic landscape of different aged vegetation (Figure 1), largely for nesting and feeding ground‐nesting game birds, namely, the Red Grouse (*Lagopus lagopu*s). In lowland heath, prescribed burning has been used alongside grazing of livestock for centuries to create a mosaic habitat and continues to be used to remove invading seedlings encroaching on rare habitat and to produce fresh growth.

These prescribed fires in the United Kingdom are applied in the winter and spring months only, within a legally defined window of 1 October to 15 April in upland sites and 1 November to 31 March for lowland sites (DEFRA, 2014). These time periods are determined, in part, to minimize wildfire risk in respect to fire weather conditions and also to avoid disturbing ground nesting birds that varies in timing with the onset of spring across the UK. “Quick, cool burns” are recommended to be used by The Heather and Grass Burning Code in England (DEFRA, 2007) and guide burn practitioners to only “remove the dwarf shrub canopy but leave behind a proportion of stick and try not to damage the moss or litter layer or expose the bare soil surface” (DEFRA, 2007, p. 18; Figure 1). While there is still variation in the severity of management fires (Glaves et al., 2013), in general, these quick, cool‐burning fires (rapidly spreading fire lines of low intensity), have less‐severe effects on plants than wildfires, with less biomass consumed, lower heat‐induced mortality, and negligible damage to their substrate with no burning into soil organic layers (Maltby et al., 1990; Allen et al., 2013). The rotational frequencies of these fires in UK heather‐dominated peatlands are typically 26.5 years on average, but varies spatially, with an average interval of 11.7 years in the North Yorkshire Moors and 25 years in the Peak District (Glaves et al., 2013).

### Plant species selection

To generate a list of native UK plant species for trait assessment, we selected those listed in the Joint Nature Conservation Committee (JNCC) National Vegetation Classification (NVC) volume of mire and heath species, which includes community codes M1‐M38 for mires and H1‐H22 for heaths (Rodwell, 1991). To collate a list of species for assessment, we first filtered the full list for all community codes (M1–M38 and H1–H22) to remove species that were listed more than once. Not all habitats within heathland and mires will be fire prone due to either their waterlogged nature (too wet to burn) or their presence on nonburnable substrates (e.g., rock ledges); hence, we removed all habitat codes that included bog pool communities, fens, soakaways, springs, flushes, rills and streambanks, rock ledges, and sand dunes and those for species that were constrained to calcareous grassland. The final list included 170 vascular plant species and 91 bryophyte species.

### Identification of fire‐adaptive traits

To assess these species, we first identified traits that support plant fitness under recurrent fire, drawing upon the large and well‐established literature on adaptive plant traits under different fire regimes across the world. In the last decade, there have been several important and thorough reviews that synthesized this literature and highlighted traits that are widely recognized as being key to fire responses (e.g., Clarke et al., 2013; Lamont et al., 2020; Pausas and Keeley, 2023).

Table 1 lists the plant traits that can inform us of a plant's response to fire and which may be readily available in species descriptions or large plant trait databases. We assessed the traits of the short‐listed species as to whether they allow plants to resist the effects of fire or persist after fire (via resprouting, recruitment from on‐site seed banks or recruitment from off‐site seed sources; Table 1). A single species can exhibit resistance and persistence traits, such that it is possible for a species to fit all categories (Rowe, 1983). In contrast, species that have none of these traits may be vulnerable to fire‐driven losses. Definitions for these groupings are listed in Table 1.

**Table 1:** Classification of adaptive traits of plants in frequently burned landscapes.

Classification	Strategy	Relevant traits	Ecological function
Resistance (capacity for aboveground plant biomass to resist the effects of fire)	Thermal insulation to fire	Bark thickness, the rate of bark thickening during growth	Protects vulnerable stem tissue from the effects of fire
	Buds positioned above the flaming zone	Presence of aboveground buds, height of buds above the ground, speed of vertical stem growth, aerial resprouting after disturbance	Protects the aboveground budbank from the effects of fire
	Low plant flammability	Biomass with high moisture content, very dense biomass	Protects biomass from the effects of fire
Persistence (capacity for plants to regenerate post‐fire after the death or top‐kill of standing individuals)	Resprouting after fire	Presence of belowground or aboveground buds, positioning of buds, number of buds, depth of buds below soil surface, protection of buds by aboveground biomass, clonal spread, presence of clonal growth organs (e.g., stolons, rhizomes), presence of belowground storage organs (e.g., bulbs, culms), basal resprouting after disturbance	Permits top‐killed individuals to persist post‐fire
	Recruiting from a seed bank that survives the effects of fire	Seed production, seed longevity, seed dormancy, seed tolerance of heating, heat‐stimulated germination, smoke‐stimulated germination, light‐stimulated germination, post‐fire seedling emergence, fire‐stimulated flowering	Permits fire‐killed populations to regenerate after fire from on‐site seed bank
	Recruiting from seeds dispersed into burnt area	Seed bank size and viability, seed dispersal ability	Permits fire‐killed populations with no viable on‐site seed bank to regenerate after fire
Avoidance (capacity of individual to avoid the effects of fire)	Living in areas unlikely to burn (‘refugia’)	Preference for low‐flammability parts of burned landscapes (e.g., in flushes, stream sides)	Permits individuals to avoid fire effects in space

It was not possible to assess the presence of avoidance traits here. The ability to avoid fire, by residing in fire‐protected microhabitats (the refugia strategy sensu Pausas, 2019), was not easy to judge, with the approach taken that focused on habitats where fire is a frequent disturbance.

### Literature search

For all 170 vascular plants, we performed two searches to identify and describe traits relevant to their responses to anthropogenic fire.

In the first set of searches, we queried three sources for species‐specific trait and fire response information. The first was the British Ecological Society's Biological Flora of Britain and Ireland Database (https://www.britishecologicalsociety.org/publications/journals/journal-of-ecology/biological-flora-database/), which contains biological and ecological descriptions of many plant species and their distribution in the British Isles. In addition, the Fire Effects Information System (https://www.feis-crs.org/feis/) provided by the United States Department of Agriculture (USDA) Forest Service, and the BROT database (Tavşanoğlu and Pausas, 2018; https://www.uv.es/jgpausas/brot.htm) were queried for fire‐related trait information about specific species. Although these latter two databases are for plant communities in other parts of the world (USA, Mediterranean Basin), they describe plant traits and responses to fire for many species found in the United Kingdom, too.

This first set of database searches was complemented by searching the wider academic and grey literature for species‐specific trait information. It was important to include the grey literature (e.g., government reports, ecological survey results), which is potentially a significant source of information about plant species responses to fire that would be missed by focusing solely on the academic literature. We therefore searched Google, Google Scholar, and Web of Science in relation to all the potential adaptive traits for each species. To query species responses to fire, we also used search terms such as “fire ecology”, “fire adaptations”, and “fire effects” for each species.

The searches for bryophyte fire responses were approached differently because initial searching indicated that data were much more rarely reported (compared to vascular plants), especially to the species level. Rather than search for information for every species or genus of bryophyte listed, we used more general search terms with the aim to pull out as much literature as possible that might describe bryophytes and fire. We started the search with the USDA Forest Service's Fire Effects Information System, which produced information for six mosses and one liverwort. We then turned to the web page search engines noted previously and searched basic terms such as “moss fire”, “sphagnum fire”, etc. Traits were also searched such as “sphagnum spore banking”, “moss spore germination heat”, “moss spore germination smoke”, etc., which identified useful literature.

Seed or spore dispersal distances were assessed for each species to evaluate their potential to recruit from off‐site seed sources. Low dispersal—due to nonspecific mechanisms (e.g., barochory) or reported distances under 5 m (dispersal classes 1 and 2; sensu Lososová et al., 2023)—was considered indicative of limited off‐site recruitment ability. We also searched for data relating to the timing of life history events for each species, specifically longevity (e.g., annual, biennial, perennial life histories), age at sexual reproduction, and phenology (e.g., time of flowering), which are important in understanding the impacts of fire frequency and timing on plant fitness (Zedler, 1995; Keeley et al., 1999; Enright et al., 2015). In addition, we searched for information about the conservation status of species in the United Kingdom, which is useful for making land management decisions based on the species present. For each taxon, we determined if they were identified as (1) a UK Biodiversity Action Plan (BAP) priority species (identified as the most threatened and requiring conservation action) and/or (2) a protected species according to the Wildlife and Countryside Act 1981 or the EU Habitats Directive (as listed at https://www.gov.uk/government/publications/protected-plants).

Overall, we reviewed ~480 literature sources (all accessed between January 2024 and March 2025) to describe the traits of native species and score them based on the presence or absence of traits relating to fire resistance and persistence. For all identified species, we described the fire effect (i.e., mortality or top‐kill), key traits, fire response (i.e., population‐scale effects), life history timing, and conservation status. For field‐based observations and experimental data, we included relevant contextual details whenever possible (e.g., location, fire type, and severity). When sources disagreed, we incorporated information from all available sources. However, classification of strategies was based on clear evidence of a taxon's ability to exhibit that trait. For example, a species capable of resprouting after a low‐severity fire but not after a high‐severity fire was classified as persisting via resprouting. We include details on the source (academic literature or grey literature) and nature (empirical evidence or expert opinion) of trait information provided in the database.

## RESULTS

### Database coverage

The database produced, which we have called the HeMi‐FiRe database (short for heath and mire plant fire response database; available in Appendix S1 and online at https://www.ukplantfiretraits.org/) incorporates information on fire‐adaptive traits and fire responses of a diverse set of native UK plant species, incorporating vascular and nonvascular plants and a wide range of growth forms (e.g., mosses, ferns, herbs, shrubs, trees). Of the taxa represented, the traits of 118 are described at the species level, and a further 35 are described at the genus or clade level (grouped when there were multiple functionally similar species of the same genera/clade that are likely to share similar traits and respond to fire in a similar way). Most taxa in the database are flowering plants from 38 angiosperm families (Table 2).

**Table 2:** Taxonomic summary of the heath and mire plant fire response (HeMi‐FiRe) database presented here.

Group	Family	Coverage	Example species
**Vascular plants**			
Angiosperms	Apiaceae	3 species	*Daucus carota*
	Asteraceae	10 species, 3 genera	*Achillea millefolium*
	Betulaceae	4 species	*Betula pendula*
	Campanulaceae	3 species	*Campanula rotundifolia*
	Caprifoliaceae	2 species	*Valeriana officinalis*
	Carophyllaceae	2 species, 2 genera	*Lychnis flos‐cuculi*
	Cistaceae	1 species	*Helianthemum nummularium*
	Convolvulaceae	1 species	*Cuscuta epithymum*
	Cornaceae	1 species	*Cornus suecica*
	Cupressaceae	1 species	*Juniperus communis*
	Cyperaceae	5 species, 3 genera	*Eriophorum vaginatum*
	Droseraceae	1 species	*Drosera rotundiflolia*
	Ericaceae	10 species, 3 genera	*Calluna vulgaris*
	Fabaceae	7 species, 3 genera	*Ulex europeaus*
	Fagaceae	1 species	*Quercus robur*
	Gentianaceae	1 species	*Centaurium erythraea*
	Geraniaceae	2 species	*Geranium sanguineum*
	Hypericaceae	3 species	*Hypericum perforatum*
	Juncaceae	2 genera	*Juncus* spp.
	Juncaginaceae	1 species	*Triglochin palustris*
	Lamiaceae	7 species	*Prunella vulgaris*
	Lentibulariaceae	1 genus	*Pinguicula* spp.
	Linaceae	1 species	*Linum catharticum*
	Onagraceae	1 species	*Chamaenerion angustifolium*
	Orchidaceae	6 species, 1 genera	*Dactylorhiza* spp.
	Orobanchaceae	3 species, 1 genus	*Euphrasia* spp.
	Oxalidaceae	1 species	*Oxalis acetosella*
	Plantaginaceae	2 species, 1 genus	*Digitalis purpurea*
	Poaceae	8 species, 6 genera	*Molinia caerulea*
	Polygalaceae	1 genus	*Polygala* spp.
	Polygonaceae	1 species, 1 genus	*Rumex* spp.
	Primulaceae	2 species, 1 genus	*Primula* spp.
	Ranunculaceae	1 species	*Ranunculus acris*
	Rosaceae	7 species, 2 genera	*Potentilla erecta*
	Rubiaceae	1 species, 1 genus	*Galium aparine*
	Salicaceae	1 species, 1 genus	*Salix repens*
	Urticaceae	1 species	*Urtica dioica*
	Violaceae	1 genus	*Viola* spp.
Gymnosperms	Pinaceae	1 species	*Pinus sylvestris*
Ferns	Equisetidae	1 genus	*Equisetum* spp.
	Polypodiidae	3 species, 1 genus	*Pteridium aquilinum*
Club mosses	Lycopodiaceae	1 clade	*Lycopodiaceae* spp.
**Nonvascular plants**			
Mosses		8 species, 1 genus	*Sphagnum* spp.
Liverworts		1 species	*Marchantia polymorpha*

Searching the published literature and trait databases for information on adaptive traits for the species of interest here was highly successful; we were able to find relevant trait information for all taxa studied here except one (*Loiseleuria procumbens*). Furthermore, for 109 taxa, we found information on fire‐adaptive traits and how they function in relation to fire (i.e., relating a trait to the ability of a species to resist or persist). For the remaining taxa, relevant trait data were found but not explicitly related to fire responses.

### Fire‐adaptive traits

**Figure 2:**
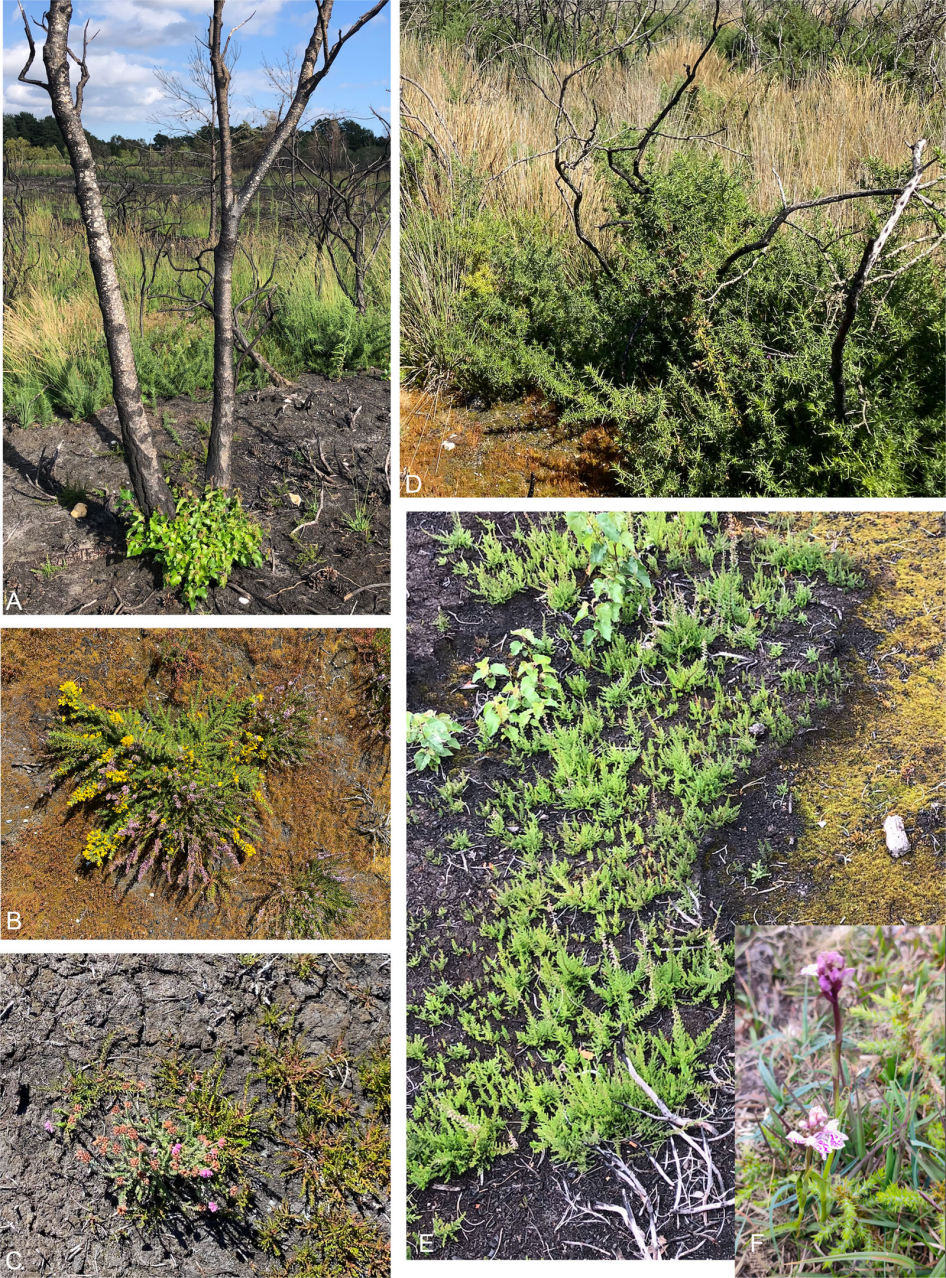
Example of fire‐adaptive traits of some heath and mire species. Photos A–E were taken after a wildfire at Ferndown Common, Dorset, UK. (A) *Betula pendula* reshooting from basal buds. (B) *Calluna vulgaris*, *Ulex gallii*, and (C) *Erica tetralix* regrowing from soil seed banks. (D) Vegetative regrowth of *Ulex europeaus*. (E) *Betula pendula* and *Calluna vulgaris* regrowing from soil seed banks. (F) *Dactylorhiza maculata* growing after a prescribed fire in the New Forest. Photo credits: (A–E) C. Belcher, (F) T. Jeffrey.

Of the UK heath and mire plant species we assessed, 97% of the taxa host fire adaptive traits. Eight of the taxa possessed fire resistance traits, while 118 had traits relating to an ability to resprout after fire. Traits linked to post‐fire recruitment from a surviving on‐site seed bank were found for 124 species, and recruitment from seed dispersed into burnt areas for 110 taxa. Only four species lacked traits that would aid their survival during or after fire in frequently burned landscapes. Overall, most heath and mire species were found to have fire‐adaptive traits that support their fitness and ability to survive fire (see Figure 2 for photos of some examples of these traits).

### Resistance

Only eight of the 153 taxa studied here are predicted to resist the effects of fire, with top‐kill or mortality commonly observed (or predicted when direct observation was lacking). Some of the tree species studied here have the potential to reach a stage where they can resist the effects of fire. For example, the English oak (*Quercus robur*) can take 10–20 years to grow tall enough to reach a height where leaves and buds are held above the flaming zone of a surface fire and have developed sufficiently thick bark to prevent damage (Jones, 1959). Fires that occur before that relatively resilient stage will cause top‐kill in this species, followed by resprouting. Scots pine (*Pinus sylvestris*) is a non‐resprouting tree in which young individuals are killed by surface fire, but those that have had time to develop a thick lower bark have a much higher likelihood of surviving (Linder et al., 1998).

In nonvascular plant species, resistance traits were present in mosses of wet environments. These have resistance to fire by being highly nonflammable due to their high moisture content (e.g., three *Sphagnum* species exhibited less than 25% cell damage on exposure to 125°C; Noble et al., 2019).

### Persistence through resprouting

The majority of the plant taxa studied here possessed traits indicative of likely ability to persist via resprouting after fire (118/153). Traits associated with the ability to resprout include buds above, at or below the soil surface, deep roots, lateral spread via rhizomes or stolons, belowground storage organs, and protective structures (e.g., basal buds that are sheathed by leaf bases in many grasses, such as *Agrostis* spp., or by a rosette of basal leaves, as in *Viola* spp.). For example, the four *Erica* species native to the United Kingdom can resprout aboveground, with recovery via epicormic buds on surviving stems after low‐severity fire (Bannister, 1965, 1966). Aerial resprouting in other species is not reported in the literature, but is likely possible in other woody species such as western gorse (*Ulex gallii*; C. Belcher, personal observation). In nonvascular plant species, such as *Pleurozium schreberi* and *Aulacomnium palustre*, recovery and reproduction after fire was possible from unburned gemmae, paraphyses, and gametophyte fragments (Tesky, 1992; Fryer, 2008).

### Persistence through recruitment from an on‐site seed bank

For 124 of the taxa studied, we found evidence in the literature that suggested they are able to endure recurrent fire through the regeneration of a population from on‐site seed or spores. Many species that establish seedlings after fire produce large viable soil‐stored seed banks, achieved through high seed production, high seed longevity or both (i.e., increasing the likelihood of viable seed being present).

In 20 of the taxa studied here, recruitment from seeds or spores may be enhanced by fire. Eighteen of these species showed evidence of heat‐stimulated germination, and 12 species exhibited smoke‐stimulated germination (Table 3). Heat‐stimulated germination was often associated with seed physical dormancy, such as a hard seed coat (e.g., gorse species [*Ulex* spp.]; Hossaert‐Palauqui, 1980; Allchin, 1998; Hanley, 2009), while smoke‐stimulated dormancy was more associated with physiological seed dormancy (e.g., grass species). However, these relationships are complicated by species with both heat‐ and smoke‐stimulated germination or that have multiple dormancy mechanisms (e.g., the double dormancy of blackberry [*Rubus fruticosus*] and St John's wort [*Hypericum* spp.]). In addition to direct fire‐stimulated germination (i.e., via heat or smoke), the germination of species can also be enhanced by the high‐light post‐fire environment (e.g., common foxglove [*Digitalis purpurea*]; Olano et al., 2002; Måren and Vandvik, 2009).

**Table 3:** UK heath and mire species with evidence of heat‐ and/or smoke‐stimulated germination.

Species/Genus	Heat stimulation	Smoke stimulation	References
*Calluna vulgaris*	Yes	Yes	Måren et al. (2010); Vandvik et al. (2014)
*Erica cinerea*	Yes	Not tested/no data	Bannister (1965); Mallik et al. (1984)
*Erica tetralix*	Yes	Yes	Mallik and Gimingham (1985); Bargmann et al. (2014)
*Erica ciliaris*	Yes	Not tested/no data	González‐Rabanal and Casal (1995); Rose et al. (1996)
*Erica vagans*	Yes	Not tested/no data	Obeso and Vera (1996)
*Vaccinium vitis‐idea*	Yes	Not tested/no data	Mallik and Gimingham (1985)
*Vaccinium myrtillis*	Yes	Not tested/no data	
*Genista anglica* and *Genista (Cytisus) scoparius*	Yes	Yes *(C. scoparius)* no data *(G. angelica)*	Tarrega et al. (1992); Bossard (1993); Rivas et al. (2006); Hanley (2009); Cruz et al. (2020)
*Ulex europaeus*	Yes	Not tested/no data	Rolston and Talbot (1980); Allchin (1998); Hanley (2009)
*Ulex gallii*	Yes	Not tested/no data	
*Ulex minor*	Yes	Not tested/no data	
*Helianthemum nummularium*	Yes	Not smoke stimulated	Poschlod et al. (2011); Martinez‐Baniela et al. (2016)
*Rubus fruticosus*	Yes	Not tested/no data	Ainsworth and Mahr (2004)
*Potentilla erecta*	Not mentioned	Yes	Bargmann et al. (2014)
*Hypericum perforatum/pulchrum*	Yes	Not tested/no data	Mallik and Gimingham (1985); Sampson and Parker (1930)
*Vicia cracca*	Yes	Not tested/no data	Ruprecht et al. (2016)
*Sphagnum fuscum*	Yes	Yes	Yusup et al. (2022)
*Sphagnum angustifolium*	Yes	Yes	
*Sphagnum squarrosum*	Yes	Yes	
Grasses: *Agrostis canina*, *Mollinia caerulea*, *Eripophorum angustifolium*, *Deschampsia flexuosa*	Not mentioned	Yes	Bargmann et al. (2014); G. Eyre, Moorland Conservationist, Derbyshire, personal communication

In the 24 non‐resprouting taxa that rely on recruitment from seed for persistence (e.g., species of the Campanulaceae [*Lobelia urens, Jasione montana*], Orobanchaceae [*Euphrasia* spp., *Melampyrum pratense*] and Plantaginaceae [*Veronica* spp.]), high seed production and longevity are frequently linked to traits that make plant survival through fire unlikely (lack of belowground buds, short lifespan [e.g., annual or biennial life history], and shallow root systems). Regeneration of fire‐killed nonvascular plant species is associated with traits including spore banking, extended spore viability (e.g., half‐life of 5–10 years in *Sphagnum* species; Clymo and Duckett, 1986), and tolerance of spores to heating (Yusup et al., 2022). Fire has also been shown to have a stimulatory effect on spore germination, and we found evidence that suggests three *Sphagnum* moss species have spores that are stimulated by heat and smoke (Table 3; Yusup et al., 2023).

### Persistence through seed dispersal

Of the assessed taxa, 110 were found to possess high dispersal ability enabling colonization of burned areas from seeds or spores that are produced off‐site. Such ability was associated with long‐distance dispersal mechanisms such as anemochory and zoochory. For example, species that rapidly colonize burned areas often have light‐weight, wind‐dispersed seeds that can travel considerable distances, increasing the likelihood of reaching a burned area for colonization (e.g., the plumed seeds of post‐fire invader rosebay willowherb *Chamaenerion angustifolium*]; Archibold, 1980; Myerscough, 1980).

For non‐resprouting species without a fire‐resistant seed bank, persistence in frequently burned landscapes relies on their capacity to recolonize from off‐site populations. From the literature, there were two examples of species that rapidly colonize recently burned areas using only light‐weight, wind‐blown spores from unburned locations—fire moss (*Ceratodon purpureus*) and common liverwort (*Marchantia polymorpha*) (Ryömä and Laaka‐Lindberg, 2005). These early pioneer species dominate after high‐severity fires (Tesky, 1992), but are unlikely to be favored by management burns, which are likely too low in severity to provide the bare landscape that the spores require for colonization.

### Species lacking fire‐adaptive traits

We found four taxa that lacked traits that would aid their survival during, or recovery after, fire in frequently burned landscapes (e.g., no or very low ability to resprout, poor seed‐banking ability). These species are phylogenetically and functionally diverse, comprising a shrub (*Juniperus communis*), herb (*Neottia cordata*), moss (*Hylocomium splendens*), and club mosses (multiple species).

The two angiosperm species, common juniper (*J. communis*) and the lesser twayblade (*N. cordata*), are highly sensitive to fire. Common juniper has very high mortality (e.g., 70–100%, Faliński, 1998), even under low‐severity fire (Mallik and Gimingham, 1985; Clifton et al., 1997), and lacks an effective soil seed bank (García et al., 1999; Lloret and Vilá, 2003). The lesser twayblade, an orchid of upland bogs, declines under burning as conditions become unsuitable with loss of the subshrub canopy and moss layer (Glaves et al., 2013). While its seeds may survive fire in the soil, population recovery will only happen when suitable conditions return (Lee et al., 2013).

Clubmosses (e.g., *Lycopodium clavatum*, *Lycopodiella inundata*, *Huperzia selago*) also likely lack fire‐adaptive traits. While data on UK‐based species is limited, studies on a North American club moss (*Lycopodium annotinum)* suggest individuals are generally killed by fire, except in quick, low‐intensity burns where rhizomes survive (Eriksson, 1989). UK club mosses have surficial rhizomes, making them particularly vulnerable (Coupe et al., 1982), and their highly flammable spores do not aid post‐fire recovery (Matthews, 1993).

Of the nonvascular plant taxa assessed here, trait information for the glittering wood moss (*H. splendens*) suggests this species experiences high mortality through fire, with populations taking many years to recover (Burch, 2008). Unlike other moss species assessed here, glittering wood moss becomes highly flammable in low humidity, and its long recovery after fire suggests its spore‐banking and colonization capabilities are poor (Tesky, 1992).

## DISCUSSION

By searching the available literature, we collected data on a range of traits considered adaptive under frequent management burns for a large number of native plant species in fire‐managed ecosystems in the United Kingdom. In addition to information on adaptive traits, we also found substantial data on specific plant responses to fire, largely in databases that catalogued these responses for other fire‐prone ecosystems around the world (e.g., FEIS database, BROT database), which included the same or closely related species to those studied here. The HeMi‐FiRe database (Appendix S1) represents a considerable step forward, by providing a knowledge base for understanding and predicting how a changing fire regime, such as the cessation of prescribed burns and increasing wildfire risk, will impact these temperate heath and mire plant communities. As such, we hope this resource will be useful for land managers and conservationists when designing management regimes in heath and mire ecosystems. Such an approach could be easily implemented for other ecosystems where information on plant responses to fire is needed but currently lacking.

As with all trait‐based approaches, there is an extrapolation between traits and their function. Here, we made assumptions about how traits influence plant function under frequent fire, for example, that the presence of belowground buds supports vegetative regeneration after fire, but such assumptions may not be correct for all species. However, for species with reliable data on both traits and responses to fire, there is good agreement between the assumed performance of traits and the response (e.g., to continue the previous example—many species that have belowground bud banks are reported to resprout following fire). In addition, by trying to bring together information on many traits, we can get a more complete understanding of trait performance (e.g., not only the presence of a bud bank, but also bud depth, number, and protective structures enabling post‐fire resprouting). By reporting species‐level traits and responses to fire, we do not acknowledge the intraspecific variation that may occur and which can be considerable (e.g., Moreira et al., 2012; Simpson et al., 2019), although in the few cases where details were given about variation across species, they have been recorded in our database. We suggest that intraspecific variation should form an area of future research. However, our species‐level trait approach provides a broad understanding of species' ability to persist under recurrent fire.

### Management implications

Many heathlands in the United Kingdom are maintained by human‐driven disturbance, such as prescribed burns or grazing of livestock, which prevent them from reaching later successional stages (e.g., scrub or woodland; Dimbleby, 1962). There has been a substantial decline in the extent of heathlands across the UK in the last century, with reduced use of land management practices, including fire, being a contributing factor, alongside land development and afforestation (Farrell, 1993). This loss of heathland has signaled an urgent need for conservation efforts to protect remaining areas of these habitats, especially given that the United Kingdom contains a significant proportion of their global extent e.g., three quarters of the world's remaining moorlands were described as being in the UK (Allen et al., 2016). Lowland heath in particular has substantially declined; with just one sixth of the lowland heath present in 1800 in England noted as remaining in 2015 (JNCC, 2015). Given the important role of human‐driven fire in the creation and maintenance of heathlands in the past, and the loss of areas of heathland when burn management stops, fire will likely be an essential tool in maintaining these habitats into the future. Our finding here that most plant species in these landscapes possess adaptive traits in relation to frequent anthropogenic burning supports this view.

For species or populations that have smoke‐ or heat‐stimulated germination (Table 3), fire will likely be needed to maintain populations by increasing the recruitment of individuals from the seed bank. For example, *Calluna vulgaris* appears to have two ecotypes that show evolutionary differentiation in germination depending on whether or not they have been exposed to long‐term anthropogenic burning regimes (Vandvik et al., 2014). Smoke‐induced germination is exhibited only in *Calluna* populations in anthropogenically managed coastal heathlands in Norway, where smoke treatment of seeds increases both the rate and likelihood of successful germination, but not in populations not subject to frequent fire (Vandvik et al., 2014). In the United Kingdom, the smoke‐stimulated form has been found in the Peak District National Park (Game and Wildlife Conservation Trust, 2020), but germination responses elsewhere have not been tested. Therefore, it is suggested that populations of *Calluna* with smoke‐stimulated germination, i.e., the “smoke‐stimulated” *Calluna* ecotype, and other heat‐ and smoke‐stimulated species will be at risk if management burn regimes cease (Vandvik et al., 2014) (although this topic has not widely been explored). A suggested alternative to management burns for conserving species that have smoke‐stimulated germination is the application of aqueous smoke solution or smoking the soil under smoking tents (van Staden et al., 2000). However, without removal of standing vegetation, seedlings may fail to establish without the open microsites that are created by fire (Måren and Vandvik, 2009).

Another group of plants with a level of dependency on anthropogenic fire are early‐successional, shade‐intolerant species. With increasing time since fire, members of this community may be lost or decrease in abundance as competition from later‐successional species intensifies and shading increases (e.g., species of the Asteraceae and Orobanchaceae families, mosses such as *Polytrichum juniperinum*). For example, in a UK lowland heath community subject to prescribed fire, 19 early‐successional plant species were only found in plots that had been burned less than 10 years previously (Smith et al., 2023). Similarly in heathland bryophyte communities, of 35 species in total, only seven were found in plots burned less than 10 years before (Burch, 2009). The evidence from UK heathlands suggests that the use of fire in these landscapes is successful in maintaining populations of early‐successional species, either as part of standing vegetation (in the short term) or within the soil seed bank (in the longer term). A mosaic of different‐aged communities, as achieved by rotational burning, will likely be effective at maintaining communities of early‐successional species and their seed banks in a landscape (Schellenberg and Bergmeier, 2020).

Fire‐based land management decisions should consider species that may thrive under frequent burning, potentially leading to local dominance and biodiversity declines. *Molinia caerulea* (purple moor‐grass) is a prime example, rapidly expanding post‐fire through both sexual and vegetative reproduction. Its aboveground biomass regrows quickly and is stimulated by fire (Brys et al., 2005). This species readily colonizes burned areas (Taylor et al., 2001) due to its high production of wind‐dispersed seeds (Bruggink, 1993; Brys et al., 2005), with seed production and germination rates further enhanced by fire (Brys et al., 2005; Jacquemyn et al., 2005). These traits create a positive feedback loop—larger *M. caerulea* populations generate more flammable biomass, increasing fire occurrence and further promoting its spread (Jacquemyn et al., 2005). As a result, *M. caerulea* encroachment poses a significant upland conservation threat, reducing biodiversity and raising the risk of large fires (Marrs et al., 2004). Other species such as bracken (*Pteridium aquilinum*) and horsetails (*Equisetum* spp.) can also increase cover greatly when burned repeatedly (Bradley et al., 1992; Natural England, 2008), and care should be taken to avoid biodiversity loss through increased dominance of these species with highly fire‐adaptive traits.

Our identification of four plant taxa that lack fire‐adaptive traits suggests that these are highly vulnerable to even low‐severity fires. One of these species, common juniper (*Juniperus communis*), has declined substantially in its UK distribution over the last century, with excessive burning leading to destruction of adult bushes and limiting regeneration of young plants listed as a factor driving this decline (JNCC, 1999). Similarly, the lesser twayblade (*Neottia cordata*) is highly sensitive to prescribed burning (Glaves et al., 2013). While seeds of this species may survive fire in the soil seed bank, population recovery can only occur when high‐humidity conditions return (i.e., upon the recovery of the moss layer and subshrub canopy) which is unlikely in the case of frequent burn rotations (Lee et al., 2013). When managing land to support populations of these fire‐sensitive species, an appropriate management scheme must be designed to avoid fire‐driven declines by using alternative management methods or leaving unburnt patches within a landscape. The life history traits of fire‐sensitive species can help determine minimum fire‐free intervals needed for individuals to reach maturity between burns.

Prescribed burns additionally play a role in preserving heathland and mire communities by reducing standing vegetation fuels, thereby lowering wildfire risk and severity. This outcome for landscapes will be increasingly required as the frequency of wildfire increases in these ecosystems because of the drier summers under global warming (Worrall et al., 2010). Therefore, prescribed burns may be important in maintaining plant communities through the reduction in wildfire occurrence and severity.

This trait‐based database provides land managers with a practical tool to plan and assess fire management in heath and mire ecosystems. It supports predictions about which species are likely to persist, decline, or influence fire behavior under various burning regimes. By linking species traits, such as age at maturity, to fire response, managers can design fire‐return intervals that sustain key species and promote biodiversity. The database also aids restoration and conservation by identifying species suited to specific regimes and those needing protection or targeted recovery after fire.

### The origin and value of adaptive traits

The traits studied here that are adaptive under recurrent application of fire may have arisen in direct response to natural selection imposed by fire, and therefore represent an adaptation to fire, or in response to another factor, and therefore represent an exaptation to fire (Gould and Vrba, 1982). Of the fire‐adaptive traits present in the UK flora studied here, fire‐stimulated germination, found in 20 taxa, represents the clearest example of a fire adaptation because heat and/or smoke stimuli are closely linked to fire and are not associated with other disturbances. In addition, rapid recruitment enhances plant fitness under frequent fire by increasing access to resources, enhancing growth rate and shortening the time to sexual maturity and seed set (Le Maitre and Midgley, 1992; Verdú and Traveset, 2005), and is therefore likely to be under strong selective pressure. Other traits, such as the ability to resprout after fire, have clear adaptive value under recurrent fire but may have arisen because of other factors (e.g., in response to herbivory or drought). Determining whether a fire‐adaptive trait is an adaptation or exaptation is difficult (Lauder et al., 1993; Midgley and Bond, 2011) and potentially irrelevant when considering habitat management and biodiversity. Because regardless of the origin of fire‐adaptive traits, their presence in a plant indicates enhanced fitness in relation to fire. Therefore, all are important to consider when thinking about fire impacts on plant communities.

Typically, fire‐adaptive traits provide fitness benefits under a specific fire regime, not to fire per se. Therefore, species that exhibit fire‐adaptive traits can be threatened when fire regimes change. While species may be able to tolerate cool‐burning management fires, it is likely that high‐severity wildfires will cause widespread mortality and the destruction of seed banks. For example, *Arctostaphylos uva‐ursi* shows high survival through controlled burns and an ability to rapidly recover biomass afterward (Barrio et al., 1999); however, high‐severity wildfires can virtually eliminate populations that were highly abundant before fire (Hamilton and Burton, 2023). Likewise, other fire regime changes, relating to frequency and seasonality, can also significantly harm populations; for example, an increase in burn frequency can make resprouting energetically unviable if plants cannot recover sufficiently before the next fire. A few species, however, do appear to tolerate a wide range of fire regimes; horsetails (*Equisetum* spp.), for example, have rhizomes that can be as deep as 2 m underground and are not killed even by very high‐severity fires (Hitchcock et al., 1969; Parminter, 1983). Therefore, the database provided here is useful in identifying species that have traits that are beneficial under prescribed fires, but it is much less able to identify species that can persist through higher‐severity wildfire events.

Moveover, when considering plant responses to fire regime changes, it is important to consider that plant traits may exhibit plasticity, where there is capacity to alter trait values in response to environmental changes within the lifetime of an organism (i.e., not requiring genetic changes; Pigliucci et al., 2006). Trait plasticity (or phenotypic plasticity) is a major mechanism of response to environmental variability, which may allow organisms to cope with rapid environmental changes. If fire‐adaptive traits are plastic, plants may be able to alter these alongside even relatively short‐term fire regime changes and maintain their adaptive value (Mitchell et al., 2021; Kane et al., 2022). However, the plasticity of fire‐adaptive traits has not been widely explored (but see Moreira et al., 2012; Simpson et al., 2019) and will likely vary considerably between species. Furthermore, the capacity of traits to maintain adaptive value will fundamentally depend on the speed and degree of fire regime changes. Therefore, the phenotypic variability of plant species within heath and mire landscapes is worthy of more detailed future study if we are to identify those species, such as *Calluna vulgaris*, that may host fire‐adapted phenotypes or ecotypes.

## CONCLUSIONS

The database created here, which details the fire‐adaptive traits of species from heaths and mires, will provide a useful resource for those who study and manage ecosystems. Many of these traits are absolutely linked to function outside the realm of fire, but by viewing them through the lens of fire, we can better understand the fitness of species through wildfire or the application of fire to the land. Furthermore, by highlighting the species that may benefit from or even be reliant on fire for recruitment from seed, our database provides a starting point for how we might consider best maintaining these species and their long‐term population health in these habitats. Finally, beyond assessing the fitness of individual species, it is important to recognize the value of trait knowledge because trait‐based approaches allow plants to be more clearly assessed for their community interactions. Such understanding of trait function allows us to better understand plant interactions with environmental change and disturbance regimes according to their fitness and adaptability (e.g., trait plasticity). As we plan strategies to reach biodiversity and healthy habitat directives, at the same time as we build ecosystems resilient to global change, understanding trait functioning—especially in relation to fire—has never been more important.

## AUTHOR CONTRIBUTIONS

All authors conceived the ideas within the manuscript. C.M.B. and K.J.S. undertook the review of the literature required to compile the database presented. C.M.B. and K.J.S. compiled the final database. K.J.S. drafted the manuscript and all authors contributed to editing the manuscript.

## Supporting information


**Appendix S1.** The heath and mire plant fire response (HeMiFiRe) database compiled using a trait‐based approach to assess the fire responses of 153 vascular and nonvascular plant taxa in UK heath and mire ecosystems managed by fire.

## Data Availability

All data presented in this manuscript are available in Appendix S1 in the supporting information.
